# Task and Resting-State fMRI Reveal Altered Salience Responses to Positive Stimuli in Patients with Major Depressive Disorder

**DOI:** 10.1371/journal.pone.0155092

**Published:** 2016-05-18

**Authors:** Yang Yang, Ning Zhong, Kazuyuki Imamura, Shengfu Lu, Mi Li, Haiyan Zhou, Huaizhou Li, Xiaojing Yang, Zhijiang Wan, Gang Wang, Bin Hu, Kuncheng Li

**Affiliations:** 1 Beijing Advanced Innovation Center for Future Internet Technology, Beijing University of Technology, Beijing, China; 2 Department of Life Science and Informatics, Maebashi Institute of Technology, Maebashi, Gunma, Japan; 3 Department of Systems Life Engineering, Maebashi Institute of Technology, Maebashi, Gunma, Japan; 4 International WIC Institute, Beijing University of Technology, Beijing, China; 5 Beijing International Collaboration Base on Brain Informatics and Wisdom Services, Beijing, China; 6 Beijing Key Laboratory of MRI and Brain Informatics, Beijing, China; 7 Mood Disorders Center, Beijing Anding Hospital, Capital Medical University, Beijing, China; 8 Ubiquitous Awareness and Intelligent Solutions Lab, Lanzhou University, Lanzhou, Gansu, China; 9 Department of Radiology, Xuanwu Hospital, Capital Medical University, Beijing, China; Laureate Institute for Brain Research and The University of Oklahoma, UNITED STATES

## Abstract

Altered brain function in patients with major depressive disorder (MDD) has been repeatedly demonstrated by task-based and resting-state studies, respectively. However, less is known concerning whether overlapped abnormalities in functional activities across modalities exist in MDD patients. To find out the answer, we implemented an fMRI experiment and collected both task and resting-state data from 19 MDD patients and 19 matched, healthy, controls. A distraction paradigm involving emotionally valenced pictures was applied to induce affective responses in subjects. As a result, concurrent deficits were found in arousing activation during a positive task in both the reward circuit and salience network (SN) that is composed of the dorsal part of anterior cingulate cortex (dACC) and bilateral anterior insulae (AI) in only the MDD group. Subsequent amplitude of low frequency fluctuations (ALFF) and functional connectivity analyses based on resting-state data exhibited consistent alterations in the bilateral AI of MDD patients, and indicated patients’ difficulties in regulating the balance between central executive network (CEN) and default mode network (DMN) due to altered connectivity among the CEN, DMN, and SN. Our findings provide new evidence demonstrating impaired salience processing and resulting alterations in responses to positive stimuli in MDD patients. Furthermore, brain abnormalities synchronized across functional states in MDD patients can be evidenced by a combination of task and resting-state fMRI analyses.

## Introduction

Major depressive disorder (MDD) is characterized by emotional and cognitive impairments, such as, alterations in emotion processing, cognitive control, affective cognition, and reward processing [1–4]. Neuroimaging methods such as positron emission tomography (PET) and functional magnetic resonance imaging (fMRI) have provided profound insights about the neural mechanisms in MDD patients, highlighting aberrant function and interaction of cortical, subcortical, (para) limbic, and midbrain regions mediating cognition, emotion, as well as metabolism [5–7]. However, due to remarkable heterogeneity in the localization and tendency of altered activity in key regions revealed by neuroimaging approaches [3], debates regarding neural correlates underlying the pathology and cardinal symptoms of MDD still exist. Multi-modal measurement has become a powerful driver of current neuroimaging research, given its advantages in recognizing potential clinical benefits [8]. It greatly improves the reliability of results, as it overcomes the limitations of individual modalities. In the present study, a combination of task and resting-state fMRI was utilized to gain a panoramic view of neural activities in the brain of MDD patients by comparing of responsiveness to emotionally valenced stimuli and spontaneous neural oscillations when the brain is at rest.

Abnormalities in processing reward-related information is considered as one of the hallmark symptoms of MDD, which have been uncovered by reward processing tasks—where the participants are instructed to interpret pleasant stimuli displayed in differential forms (e.g., pictures [9], words [10], facial expressions [11], etc.), or to respond to monetary reward [12, 13]. Despite considerable variations in types of stimuli, a common frontostriatal network showing abnormalities across tasks has been identified in the MDD brain [14]. Specifically, MDD patients generally showed reduced activities in subcortical and limbic areas, including the caudate, putamen, thalamus, insula, amygdala, and anterior cingulate; and increased activities in the dorsolateral prefrontal cortex (DLPFC) and additional occipital areas, including the middle frontal gyrus, superior frontal gyrus, cuneus and lingual gyrus in MDD patients. Most of these regions have long been conceived as major players in reward anticipation, reward receipt, as well as responses to stimuli with positive emotional valence [15–17], given that they are the main projection areas of two distinct dopaminergic pathways, the nigrostriatal and mesocortical [18]. However, it remains unclear how dopamine neurons distinctively modulate activity in these brain areas.

On the other hand, reward processing does not represent a unitary construct, nor does it rely on a singular biological circuit [19]. Emerging evidence suggests the relationship between the biased attentional processing and altered recognition of reward or positive stimuli in MDD patients. For example, depressed patients exhibited reduced attention to positive facial emotion expressions compared with increased vigilance towards negative expressions [20, 21]. Patients with depression also showed decreased perceptual sensitivity to positive words and pictures [22]. Generally, the relative salience of stimuli determines which inputs are more likely to capture attention when the brain is constantly bombarded by stimuli. A growing body of literature has identified misappropriated stimulus-driven salience detection and altered attentional processes in patients with psychiatric disorders [23]. An insula-anterior cingulate cortex (ACC) salience network has been proposed to be responsible for the salience processing, and also to serve as a hub that enables switching of brain states from the default mode to a task-related activity mode [24, 25]. For that reason, the salience network functioning has been considered as a key to the morbid rumination—a core vulnerability in MDD brains, because the aberrant toggle system is likely to result in the imbalance between default mode and executive networks [26]. Recently, the potential role of altered salience processing in abnormal responses to rewards has also been highlighted, due to the evidence showing disrupted interactions between the insula-ACC regions and reward-related areas in psychotic patients [27]. It appears that blunted processing of incentive salience could be another candidate for explaining altered reward processing in MDD patients. However, further investigations need to be implemented to transparentize intrinsic neural mechanisms regarding how impaired salience system influences the reward processing.

In the past few years, resting-state fMRI (rs-fMRI) has been pervasively employed to investigate the spontaneous neural fluctuations of the human brain. Particularly, rs-fMRI functional connectivity based on temporal dependency of neural activation of anatomically separated brain regions enables the exploration into the overall organization of functional communication in the brain networks [28]. Therefore, the rs-fMRI functional connectivity has also been applied as a powerful method to reveal the abnormalities in intrinsic connectivity between the salience network and other cognitive or affective systems of patients with psychiatric disorders [29, 30]. For instance, prior studies documented disrupted connections from the hippocampus and amygdala to the dorsomedial-prefrontal cortex and salience-related fronto-insular operculum in MDD patients [31]. Also, altered rs-fMRI functional connectivity between the pregenual anterior cingulate cortex and the right anterior insula (AI) was found exclusively in the subgroup of severely depressed patients, compared to healthy subjects and mildly depressed patients [32]. Given that more and more studies have shown the strong synchronization of spontaneous blood-oxygen-level dependent (BOLD) oscillations and measured fluctuations in neuronal spiking [33, 34], it seems that the resting brain activities can be used as a reflection of neuronal signals. Moreover, an integration of task and resting-state fMRI is likely to enable more fine-grained analyses on psychiatric disorders by coupling altered spontaneous neural signals to the impact on brain responses to specific stimuli.

Here, we examined responses to different affective images and attentional control of MDD and healthy cohorts by employing a distraction paradigm, and combined the brain responses to affective processing tasks and the resting-state BOLD oscillations. We hypothesized that common regions showing alterations in both task and resting states would provide more reliable references to find the latent neural substrates that underlie the pathology of MDD.

## Materials and Methods

### Ethics statement

The recruitment of MDD patients for this study was approved by the Ethics Committee of Anding Hospital, Capital Medical University, Beijing. Prior to participation in the study, written informed consent was obtained from each participant, after the nature and possible consequences of the studies were explained. The fMRI experiment was approved by the Ethics Committee of Xuanwu Hospital, Capital Medical University, Beijing.

### Subjects

Nineteen, right-handed, MDD patients (8 males and 11 females) were recruited among outpatients from Beijing Anding Hospital, China, and 19 healthy controls (HC) matched for gender, age, and years of education with MDD patients were recruited from the community. Diagnostic assessments for all participants were performed by clinically trained and experienced raters (T. Tian and B. Fu) using the Mini International Neuropsychiatric Interview 6.0 (MINI 6.0) based on DSM-IV [35]. Hamilton Depression Rating Scale 17 Items (HDRS-17) was used to evaluate the clinical symptom severity of depression for only MDD patients. The 9-item Patient Health Questionnaire (PHQ-9) and 16-Item Quick Inventory of Depressive Symptomatology (QIDS) were applied to make self-reports about the depressive symptoms for both the MDD and HC groups. Anxiety level of all participants was evaluated by using the Trait Anxiety Inventory (T-AI). Participant demographics and clinical characteristics are presented in Table 1. The exclusion criteria were: (1) depressive patients with any mania episode or history of any comorbid major psychiatric illness on Axis I or Axis II; (2) concurrent serious medical illness or primary neurological illness; (3) history of head injury resulting in loss of consciousness; (4) abuse of or dependence on alcohol or other substances; (5) contraindication for MRI.

**Table 1:** Demographic and clinical characteristics of MDD patients and healthy controls. Abbreviation: HDRS-17, Hamilton Depression Rating Scale 17 Items; PHQ-9, Patient Health Questionnaire 9 Items; QIDS, Quick Inventory of Depressive Symptomatology; T-AI, Trait Anxiety Inventory.

Characteristics	MDD Patients (n = 19)	Controls (n = 19)	p -Value
Gender (male: female)	8: 11	8: 11	1
Mean age (years)	33.8 ± 10.5	33.3 ± 9.9	0.88
Education level (years)	14.1 ± 3.2	13.9 ± 3.6	0.89
HDRS-17 Total Score	15.8 ± 8.0	-	-
PHQ-9	11.3 ± 6.2	3.9 ± 3.1	0.00
QIDS	11.4 ± 5.6	4.3 ± 3.5	0.00
T-AI Total Score	51.2 ±11.6	38.1 ± 8.8	0.00

### Experimental design

Both resting-state and task-state data were collected in the present study. Prior to the start of the task experiment, a five-minute resting-state fMRI measurement with eyes open was implemented for all subjects.

The task design modifies and combines previous paradigms of distraction tasks to study both affective processing and attentional control of MDD patients [36, 37]. Three task conditions were included and presented in a block-designed pattern. Subjects were shown pictures and then required to solve mental arithmetic problems presented as overlays on the pictures. Three types of pictures, corresponding to each task condition, were applied, with positive (e.g., joyful, exciting), neutral, and negative (e.g., aversive) valences, respectively. As distractors, 2-digit simple mental addition and subtraction problems, without carrying and borrowing, were employed to avoid ceiling and floor effects. The difficulty level of such arithmetic problems was verified to be appropriate for attracting attention of both MDD and healthy subjects by performing a pre-experiment.

Each trial consisted of an emotion induction phase and a distraction phase. During the induction phase (2000 ms), a picture with a specific valence was displayed. Subjects passively viewed the picture to elicit an initial emotional response. During the distraction phase (4000 ms), subjects needed to shift attention from the picture to an arithmetic problem, and then decide whether the displayed solution was correct or incorrect by pressing two response keys using the left and right thumbs. The accuracy and reaction time of each response were recorded. Incorrect displayed solutions deviated by ± 1 or ± 10 from the correct solutions in 50% of all the trials. The frequency of occurrence of each number was balanced and the proportion of each arithmetic operation was 50% for all conditions. Neither “tie” problems (e.g., 32 + 32, 67–67) nor repeated problems were recruited. Twelve successive trials with the same task condition constituted a task block. Blocks of three conditions were mixed and counterbalanced, and every two task blocks were separated by a rest block. Data were acquired in three functional runs with a total of 36 trials for each type of task.

Affective pictures were selected from the International Affective Picture System (IAPS) which is based on normative ratings in valence and arousal [38]. The contents of positive pictures included beautiful scenery, delicious food, scenes of sports, romance, and money, with mean valence of 7.49 ± 1.54 and mean arousal of 5.36 ± 2.25. Aversive pictures, such as snakes, spiders, attacks, bloody wounds, and dead bodies were adopted for the negative stimuli, with mean valence of 2.61 ± 1.60 and mean arousal of 6.30 ± 2.14. Pictures of household items in simple contexts (e.g., a cup on a table) were used as the neutral stimuli, with mean valence of 5.01 ± 1.13 and mean arousal of 3.05 ± 1.94.

### MR data acquisition

The fMRI data were acquired with a 3.0 Tesla MRI scanner (Siemens Trio Tim, Siemens Medical System, Erlanger, Germany) using a 12-channel phased array head coil. Foam padding and headphones were used to limit head motion and reduce scanning noise. One hundred ninety two slices of anatomical images with a thickness of 1 mm were obtained using a T1 weighted 3D magnetization prepared rapid gradient echo (MPRAGE) sequence (TR = 1600 ms, TE = 3.28 ms, TI = 800 ms, FOV = 256 mm, flip angle = 9°, voxel size = 1 × 1 × 1 mm^3^). Functional images for both task state and resting-state were collected through an echo-planar imaging (EPI) sequence (TR = 2000 ms, TE = 31 ms, flip angle = 90°, FOV = 240 mm, matrix size = 64 × 64). Thirty axial slices with a thickness of 4 mm and an interslice gap of 0.8 mm were acquired.

### Data preprocessing

The preprocessing of fMRI data was performed with SPM12 software (Wellcome Trust Centre for Neuroimaging, London, UK, http://www.fil.ion.ucl.ac.uk) and REST Toolkit [39] implemented on a MATLAB platform (MathWorks, Natick, MA). The first two images of task-state and ten images of resting-state data were discarded to allow the magnetization to approach dynamic equilibrium. Functional images were corrected for slice-timing differences and realigned to the median image to correct rigid body motion. Patients with head movement exceeding 3 mm or 3 degrees and healthy subjects exceeding 2 mm or 2 degrees were rejected. The high resolution anatomical image was co-registered with the mean image of the EPI series and then spatially normalized to the MNI template. After applying the normalization parameters to the EPI images, all volumes were resampled into 3 × 3 × 3 mm^3^. Then the normalized task-state images were smoothed with an 8-mm FWHM isotropic Gaussian kernel, whereas resting-state images were spatially smoothed with a 4-mm FWHM isotropic Gaussian kernel for conventional sake. The linear detrending and band-pass filtering (0.01–0.08 Hz) were performed on the resting-state time series, followed by regressing out the mean time series of global, white matter and cerebrospinal fluid signals, to remove artifacts and reduce physiological noise.

### Functional MRI analysis

Task-state data were statistically analyzed using SPM12. After specifying the design matrix, each participant’s hemodynamic responses induced by the trials were modeled with a box-car function convolved with a hemodynamic function. The parameters for the effects of the positive task (PT), neutral task (NEUT), and negative task (NT) which displayed pictures with respective valences were estimated. Contrast images were constructed individually based on the general linear model (GLM). Due to the involvement of two factors in the present study, the group-level analysis was implemented based on a 2 by 3 factorial design with factors of “Group” (2 levels) and “Condition” (3 levels). Main effects of “Group” and “Condition” were analyzed to confirm whether differences in brain activation patterns exist between MDD patients and healthy controls (HC), and among PT, NEUT, and NT. Interaction was also examined for the two factors. Further inspections for the simple effects could be computed following a significant interaction, by which comparisons between groups under either emotion state would be allowed. Thresholds were set at a voxel-level p < 0.005, cluster size > 1242 mm^3^, corresponding to a corrected p < 0.05 as determined by AlphaSim correction.

Analyses for resting-state data were conducted by using REST Toolkit. A whole-brain amplitude of low frequency fluctuations (ALFF) map of each subject was calculated and transformed to zALFF map via a standardization process. A two-sample t-test was performed on the zALFF maps between MDD and HC groups. Resulting brain regions with significant differences were overlapped with the aforementioned brain maps that resulted from task-state analyses. Common regions with differences implicating consistence of neural alteration of MDD patients across both task and resting states, especially those attentional processing-related regions included in the salience network, were considered as seeds for the further analyses. Functional connectivity analysis was performed using a voxel-wise correlation approach based on the resting-state time series. Pearson correlation coefficients between one seed and the rest of the brain were computed voxel-by-voxel. Differences in functional connectivity between the MDD and HC groups were compared by two-sample t-tests following a Fisher’s r-to-z transformation. Thresholds were set at a voxel-level p < 0.005, cluster size > 351 mm^3^, corresponding to a corrected p < 0.05 as determined by AlphaSim correction.

Finally, in order to examine the interactions between brain functions and depressive symptoms, the mean percentage BOLD signal change acquired during the positive task condition and averaged zALFF scores of each seed were extracted by a 6 mm-radius sphere to serve as functional indicators for each subject. In addition, standardized correlation coefficients of altered connections linking the seeds and other regions revealed by the resting-state functional connectivity analysis were selected as another sign of brain abnormalities. All the values acquired by fMRI were correlated with the clinical assessment of depressive symptoms. SPSS 19.0 software (SPSS, Chicago, IL, USA) was used for the statistical analyses.

## Results

### Behavioral results

We carried out two-way analyses of variance on the accuracy (ACC) and reaction time (RT) by specifying the 3 task conditions as the within-group factor (Condition) and the 2 groups as the between-group factor (Group). In the MDD group, the average ACC was 86.84 ± 11.51% (mean ± SD) for the positive task (PT), 85.96 ± 15.94% for the neutral task (NEUT), and 85.75 ± 12.46% for the negative task (NT). In the HC group, the average ACC was 92.25 ± 7.49% for the PT, 92.98 ± 6.10% for the NEUT, and 90.94 ± 5.23% for the NT. Only the main effect of Group was significant, with the F (1, 108) = 8.897, p = 0.004. MDD patients showed significantly lower ACC than the healthy subjects.

In the MDD group, the average RT was 2461.96 ± 463.03 ms for the PT, 2467.21 ± 498.99 ms for the NEUT, and 2500.29 ± 485.92 ms for the NT. In the HC group, the average RT was 2391.31 ± 375.99 ms for the PT, 2405.70 ± 346.38 ms for the NEUT, and 2452.68 ± 384.96 ms for the NT. Neither main effect nor interaction reached significance.

### Group comparison of task-induced activation

**Fig 1:**
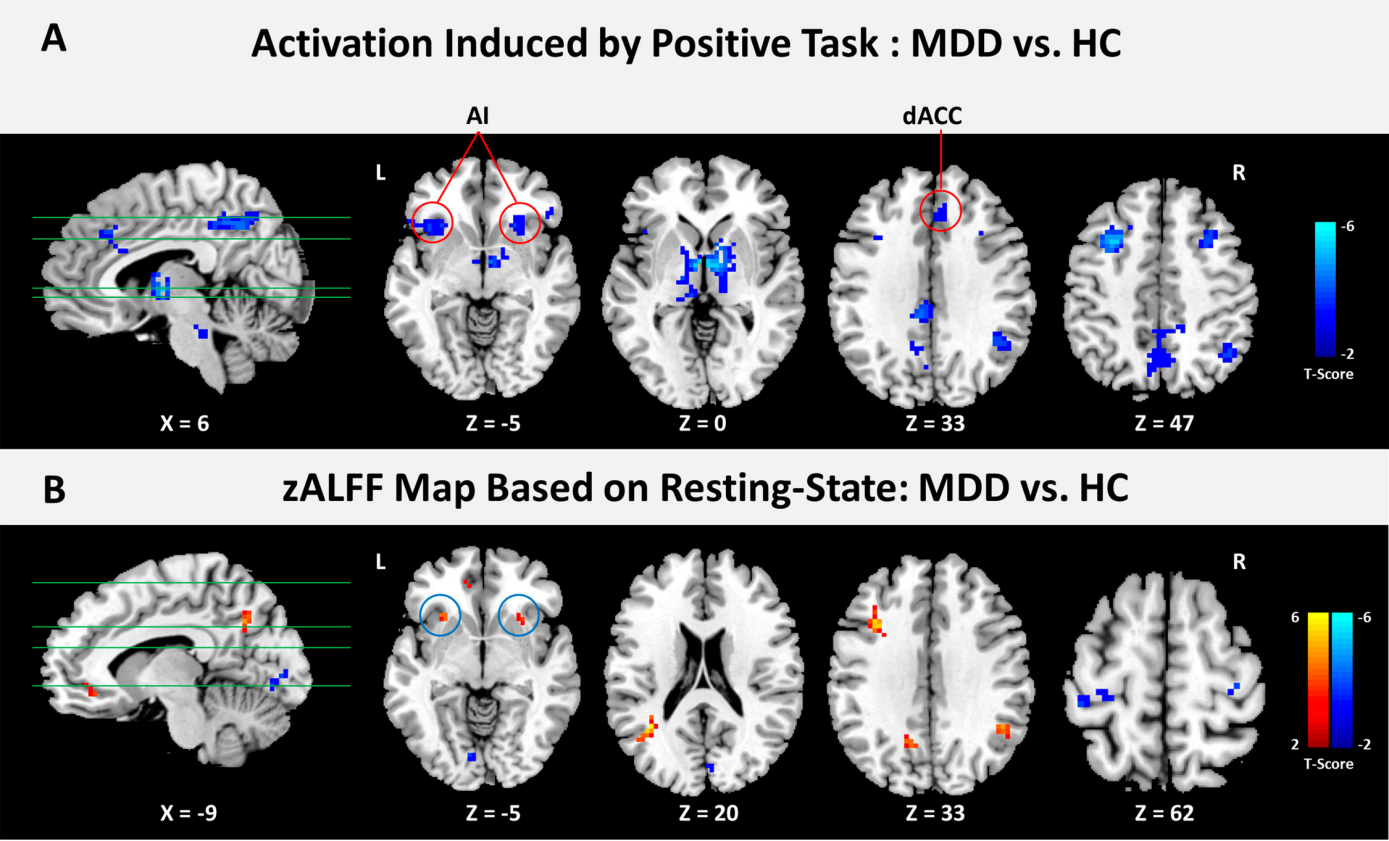
Regions showing group differences in task-induced activation and resting-based ALFF. (A) Compared with the HC group, the MDD group showed only decreased activation in the frontal-parietal, subcortical, and midbrain areas, especially in the dorsal part of the anterior cingulate cortex (dACC) and anterior parts of the bilateral insulae (AI) which constitute the salience network. (B) Comparison between the two groups demonstrated significant differences in the standardized ALFF (zALFF) values based on resting-state data. The bilateral AI showed significantly increased zALFF values in the MDD group. The color bars indicate t-values for each analysis.

The group-level analysis based on factorial design exhibited significant main effects of Group and Condition, as well as significant interaction between Group and Condition (see S1 Fig). Post hoc 2-sample t-tests were implemented to examine brain activation differences in emotional responses and attentional control between MDD and HC groups under different emotional states. In the positive condition, MDD patients showed only decreased brain activation in the left anterior insula (AI), right orbital part of the inferior frontal gyrus around the AI, dorsal part of the anterior cingulate cortex (dACC), left precuneus, bilateral angular gyri (AG), bilateral dorsolateral prefrontal cortices (DLPFC), and bilateral thalamus extending to the putamen, caudate nuclei, pallidum, and other subcortical areas (see Fig 1A). In the neutral condition, decreased brain activation was observed in the right precuneus and left DLPFC in MDD patients, compared with healthy subjects. In the negative condition, the MDD group showed a similar pattern as in neutral condition, with only decreased activation in the right precuneus and left DLPFC. All the regions with significant activation are listed in Table 2. No increased activation was found for MDD patients in either condition.

**Table 2:** Regions with decreased activation elicited by contrasts of MDD versus HC in positive, neutral, and negative tasks. Thresholds were set at a voxel-level p < 0.005, cluster size > 1242 mm^3^ voxels, corresponding to a corrected p < 0.05 as determined by AlphaSim correction. Abbreviation: IFGorb, orbital portion of inferior frontal gyrus; dACC, dorsal part of anterior cingulate cortex; DLPFC, dorsolateral prefrontal cortex; AG, angular gyrus; L, left; R, right; BA, Brodmann area.

Condition	Region	BA	Cluster	Peak MNI			T-score
				x	y	z	
Positive Task	L. Insula	47/13	87	-36	24	-12	-3.86
	R.IFGorb / Insula	47	44	42	51	-12	-4.21
	R. dACC	32	43	6	27	36	-3.24
	R.Thalamus		73	12	-3	0	-4.56
	L.Thalamus		69	-3	-3	0	-4.57
	L. DLPFC	8/6	54	-33	9	57	-4.39
	R. DLPFC	8/6	45	36	18	54	-4.47
	L. Precuneus	7	168	3	-54	42	-4.29
	L. AG	40	54	-45	-63	30	-3.40
	R. AG	40	52	42	-57	54	-3.54
Neutral Task	R. Precuneus	7	78	6	-57	39	-3.56
	L. DLPFC	8/6	50	-33	18	42	-3.52
Negative Task	R. Precuneus	7	45	3	-60	42	-3.63
	L. DLPFC	6	52	-33	6	57	-3.59

### Group comparison of zALFF values based on resting-state data

A two-sample t-test was applied to reveal differences in zALFF values between MDD and HC groups. As shown in Fig 1B, regions presenting significantly increased zALFF values in the MDD group included the left subgenual part of the anterior cingulate cortex (sgACC), left DLPFC, left precuneus, right AG, bilateral middle temporal gyri (MTG), and bilateral AI. Compared with the HC group, regions with significantly decreased zALFF values in the MDD group were found at the left postcentral gyrus, left lingual gyrus (LG), right cuneus, bilateral precentral gyri, and bilateral parahippocampal gyri / amygdalae. All the regions presenting significant differences in zALFF values between the two groups are listed in Table 3.

**Table 3:** Regions showing group differences in zALFF values. Thresholds were set at a voxel-level p < 0.005, cluster size > 351 mm^3^ voxels, corresponding to a corrected p < 0.05 as determined by AlphaSim correction. Abbreviation: sgACC, subgenual part of anterior cingulate cortex; MTG, middle temporal gyrus; DLPFC, dorsolateral prefrontal cortex; AG, angular gyrus; Parahip, parahippocampal gyrus; Amyg, amygdala; PreCG, precentral gyrus; PostCG, postcentral gyrus; LG, lingual gyrus.

Contrast	Region	BA	Cluster	Peak MNI			T-score
				x	y	z	
MDD > HC	L. Insula	13	27	-27	18	-3	6.77
	R. Insula	13	59	30	12	9	6.15
	L. sgACC	10	15	-9	36	-12	3.81
	L. MTG	39	25	-39	-57	18	6.59
	R. MTG	21	14	66	-54	9	3.74
	L. DLPFC	9	20	-36	15	30	5.31
	L. Precuneus	7	27	-9	-63	36	4.50
	R. AG	40	28	45	-51	30	7.91
MDD < HC	L. Parahip/ Amyg	28	13	-21	-9	-24	-3.51
	R. Parahip/ Amyg	28	13	18	-6	-27	-3.58
	L. PreCG	6	20	-54	0	18	-4.12
	R. PreCG	4	13	39	-24	51	-3.72
	L. PostCG	4	21	-36	-30	69	-4.34
	L. LG	18	13	-9	-81	-6	-3.77
	R. Cuneus	19	16	6	-90	27	-4.03

**Fig 2:**
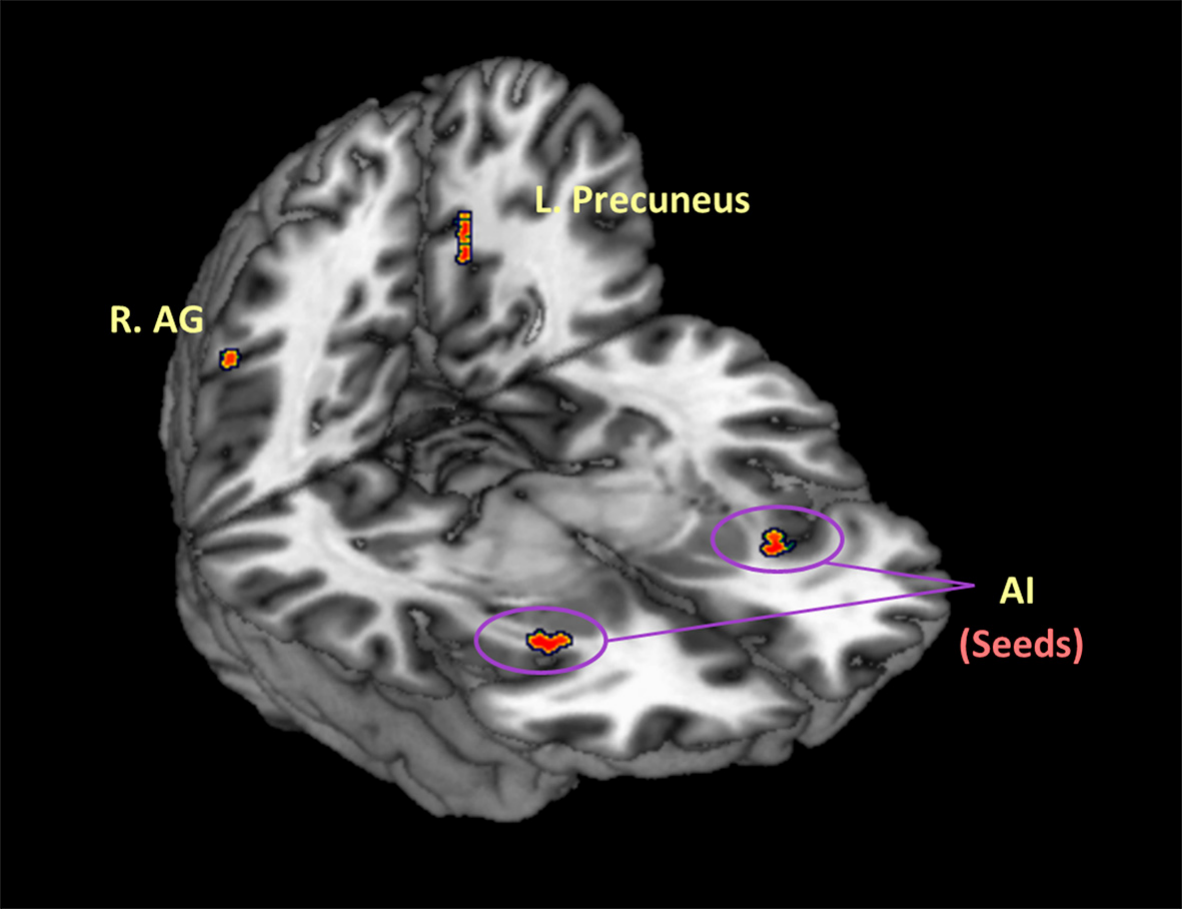
Brain regions showing synchronized abnormalities across task and resting states in MDD group. Regions with synchronized abnormalities in the MDD group are shown by overlapping alterations revealed in task-activated brain maps and resting-based zALFF map, respectively. Among the regions, the bilateral anterior insulae (AI) were determined as seeds for further analyses.

Resulting zALFF maps with group differences were overlapped onto the brain maps involving activation differences observed in task conditions. The common regions were located at the bilateral AI, left precuneus, and right AG (see Fig 2 and Table 4). According to our hypothesis proposing that altered emotion responses in MDD patients might be implicated in abnormalities in salience processing, the bilateral AI anchored at (-30, 21, -6) and (30, 15, -9) in MNI space were selected as seeds for the further analyses, in consideration of their established roles in salience detection and reward processing.

**Table 4:** Common brain regions showing abnormalities in both task and resting states in MDD group. The bilateral anterior insulae (AI) were determined as seeds for further analyses.

Region	BA	MNI Coordinates		
		x	y	z
L. Insula	13	-30	21	-6
R. Insula	13	30	15	-9
L. Precuneus	7	-12	-60	36
R. AG	40	45	-51	30

### Group comparison of resting-state functional connectivity

**Fig 3:**
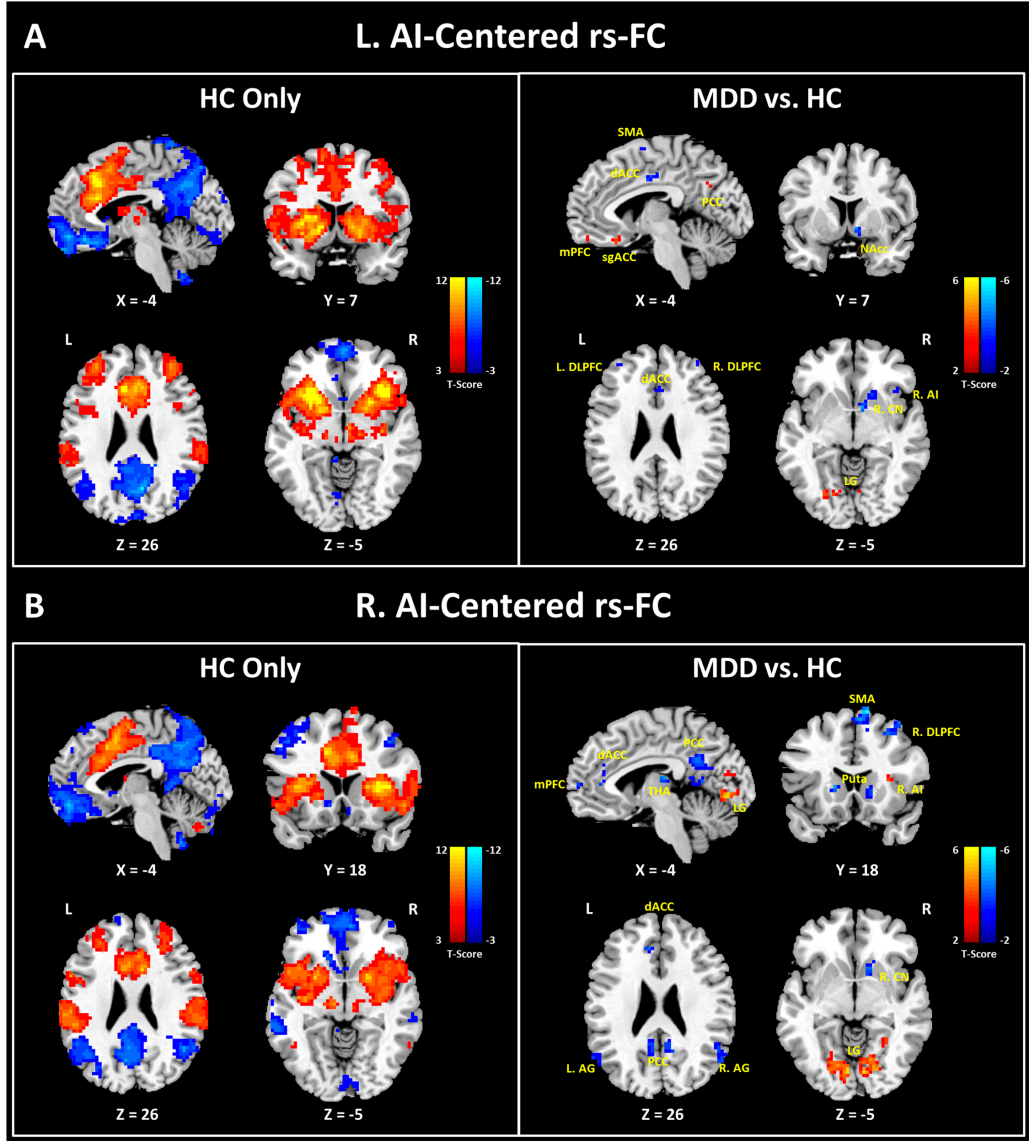
Results of resting-state functional connectivity analysis (rs-FC). Voxel-wise analyses were performed for each subject, followed by group-level t-tests. One-sample t-tests were only used for the HC data to show the intact rs-FC in the healthy state (left parts of A and B). Two-sample t-tests were used to show the altered rs-FC caused by MDD (right parts of A and B). (A) Results of L. AI-centered rs-FC analysis. (B) Results of R. AI-centered rs-FC analysis. The color bars indicate t-values for each analysis. Abbreviation: mPFC, medial prefrontal cortex; sgACC, subgenual part of anterior cingulate cortex; dACC, dorsal part of anterior cingulate cortex; SMA, supplementary motor area; PCC, posterior cingulate cortex; NAcc, nucleus accumbens; LG, lingual gyrus; DLPFC, dorsolateral prefrontal cortex; CN, caudate nucleus; AI, anterior insula; THA, thalamus; Puta, putamen; AG, angular gyrus; L, left; R, right.

Voxel-wise functional connectivity (FC) analyses revealed the Pearson correlation coefficients between the seeds and rest parts of the brain. Two-sample t-tests were used to disclose the group differences in the connectivity (see Fig 3 and Table 5). After the comparison of the L. AI-centered FC maps, regions revealing significantly increased correlation to the seed in the MDD group contained the left sgACC, left medial prefrontal cortex (mPFC), left LG, right calcarine, and left precuneus. Regions showing significantly decreased correlation included the right insula, right caudate nucleus (CN) ventrally extending to the nucleus accumbens (NAcc), right dACC, left middle cingulate gyrus (MCG), right superior parietal lobule (SPL), bilateral DLPFC and supplementary motor area (SMA). On the other hand, comparison of the R. AI-centered FC maps in MDD versus HC brains showed significantly increased correlation between the seed and the right insula, left middle occipital gyrus, bilateral LG, and bilateral superior temporal gyrus. Regions showing decreased correlation to the right AI included the right CN, left putamen, left mPFC, left dACC, right MCG, right SMA, bilateral posterior cingulate cortex (PCC), AG, and DLPFC.

**Table 5:** Regions showing group differences in voxel-wise functional connectivity based on resting-state data. Thresholds were set at a voxel-level p < 0.005, cluster size > 351 mm^3^ voxels, corresponding to a corrected p < 0.05 as determined by AlphaSim correction. Abbreviation: sgACC, subgenual part of anterior cingulate cortex; mPFC, medial prefrontal cortex; LG, lingual gyrus; NaCC, nucleus accumbens; dACC, dorsal part of anterior cingulate cortex; DLPFC, dorsolateral prefrontal cortex; MCG, middle cingulate gyrus; SMA, supplementary motor area; SPL, superior parietal lobule; STG, superior temporal gyrus; MOG, middle occipital gyrus; PCC, posterior cingulate cortex; AG, angular gyrus.

Seed (Contrast)	Region	BA	Cluster	Peak MNI			T-score
				x	y	z	
**L. AI**							
(MDD > HC)	L. sgACC	25	16	-6	24	-21	3.86
	L. mPFC	10	13	-6	51	-18	3.42
	L. LG	18	13	-27	-75	-6	3.88
	R. Calcarine	18	14	3	-78	3	3.28
	L. Precuneus	7/31	16	-3	-69	27	3.22
(MDD < HC)	R. Insula	47	20	39	18	-9	-4.70
	R. Caudate		34	18	15	-6	-4.03
	R. NaCC		30	9	6	-6	-4.70
	R. dACC	24	25	6	30	24	-3.95
	L. DLPFC	10	19	-36	48	27	-3.23
	R. DLPFC	10	15	39	51	30	-3.44
	L. MCG	24	36	-3	-9	36	-4.41
	R. SMA	6	43	12	18	57	-4.70
	R. SPL	7	16	39	-63	60	-3.99
**R. AI**							
(MDD > HC)	L. LG	18	158	-6	-81	-6	5.30
	R. LG	18	186	21	-78	-9	5.77
	L. STG	41	18	-51	-27	9	4.17
	R. STG	22	16	60	-6	0	3.53
	L. MOG	19	14	-27	-90	30	3.58
	R. Insula	13	13	30	15	12	4.27
(MDD < HC)	R. Caudate		20	15	15	-6	-4.34
	L. Putamen		14	-15	18	3	-4.7
	L. mPFC	10	20	-9	57	6	-4.38
	L. dACC	32	15	-9	39	6	-4.81
	R. MCG	24	15	3	-24	39	-3.50
	L. PCC	29	36	-6	-57	9	-3.95
	R. PCC	31	122	6	-54	30	-4.58
	L. AG	39	23	-60	-63	21	-3.78
	R. AG	39	116	51	-54	33	-4.75
	L. DLPFC	8	29	-24	27	57	-4.22
	R. DLPFC	8/6	65	30	15	48	-4.52
	R. SMA	8	61	12	18	66	-5.60

### Correlations between fMRI results and clinical data

**Fig 4:**
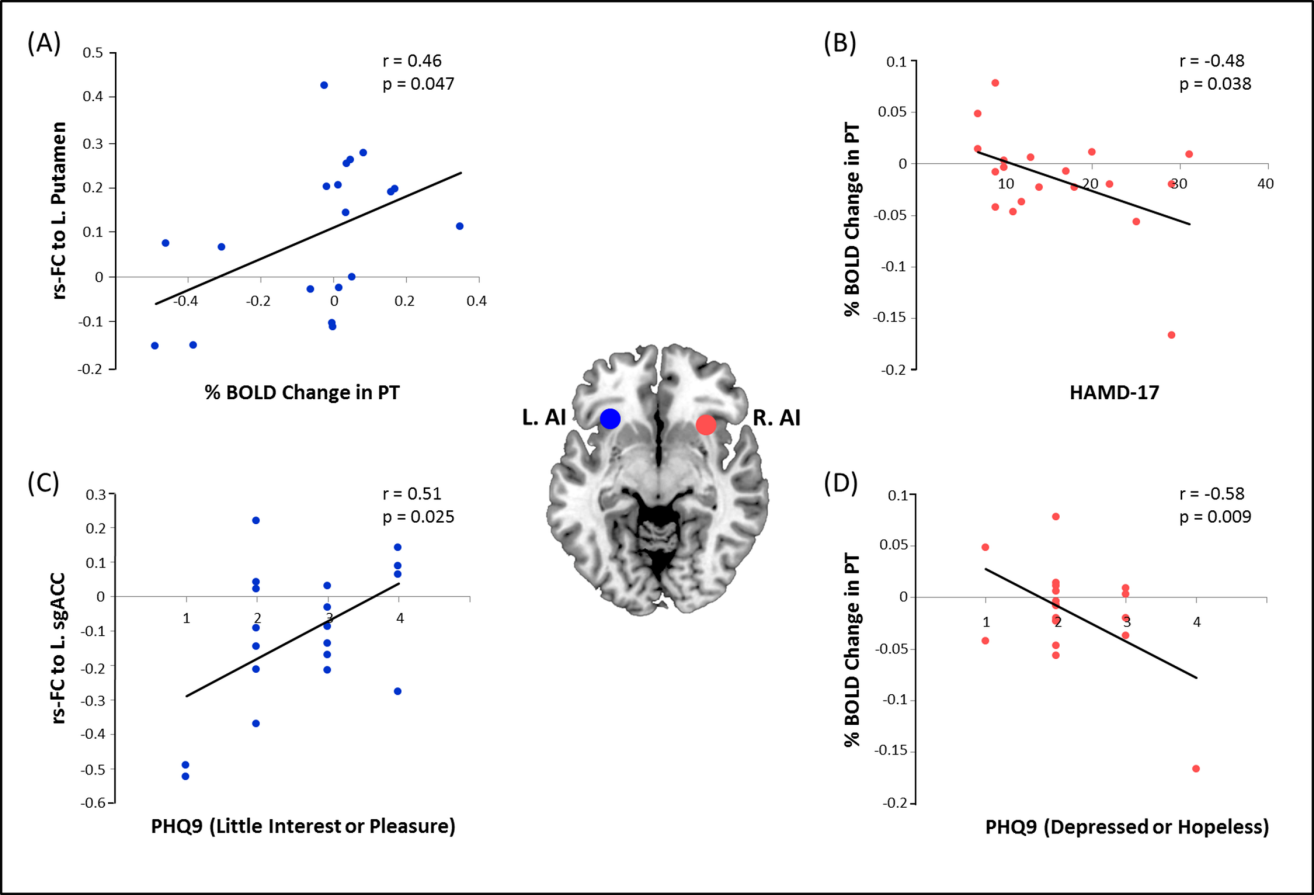
Correlations between fMRI results and clinical data in MDD group. (A) The rs-FC between the left AI and ipsilateral putamen positively correlated to the mean percentage BOLD signal change of the left AI during the PT; (B) mean percentage BOLD signal change of the right AI during the PT exhibited negative correlations with the total score of HDRS-17; (C) the rs-FC between the left AI and left sgACC positively correlated to the subscore for “little interest or pleasure in doing things” of PHQ-9; (D) mean percentage BOLD signal change of the right AI during the PT presented negative correlations with the subscore for “feeling down, depressed or hopeless” of PHQ-9. The HC group did not show any significant correlation between brain functional data and clinical scores. Abbreviation: rs-FC, resting-state functional connectivity; PT, positive task; AI, anterior insula; sgACC, subgenual part of anterior cingulate cortex; HDRS-17, Hamilton Depression Rating Scale 17 Items; PHQ-9, Patient Health Questionnaire 9 Items.

In the MDD group, the mean percentage BOLD signal change of the right AI during the positive task exhibited significant negative correlations with the total score of the Hamilton Depression Rating Scale 17 Items (r = -0.48, p = 0.038) and the subscore for “feeling down, depressed or hopeless” of the 9-item Patient Health Questionnaire (r = -0.58, p = 0.009). Further investigations based on results of resting-state functional connectivity analysis showed that the correlation coefficient between the left AI and ipsilateral putamen positively correlated to the mean percentage BOLD signal change of the left AI during the positive task (r = 0.46, p = 0.047), while the coefficient between the left AI and left sgACC positively correlated to the subscore for “little interest or pleasure in doing things” of the 9-item Patient Health Questionnaire (r = 0.51, p = 0.025). No significant correlation was found between other fMRI results and clinical data in MDD patients. In the HC group, neither the task-induced brain activities nor resting-state signals revealed significant correlation with clinical data. Results of correlations between fMRI results and clinical data are shown in Fig 4.

## Discussion

In the present study, brain activation maps resulted from affective tasks and zALFF values obtained from resting-state data of MDD patients and healthy controls were overlapped. Common regions showing group differences observed in both states were selected as seeds for guiding subsequent resting-state functional connectivity analyses. Finally, consequent multi-modal fMRI results were correlated with the symptom scores of depression and anxiety. Several alterations demonstrating aberrant executive control, ruminative, reward-related processes, especially disrupted salience system, were exhibited in MDD patients by means of cross-referencing task and resting-state fMRI data.

### Aberrant executive control, ruminative, and reward processes in MDD patients

As post hoc results of group-level analyses, only significantly decreased activation was revealed by comparing MDD patients with HC subjects in all three task conditions. Hypoactivity was found in the left DLPFC and precuneus across the three conditions in the MDD group. The left DLPFC is well-known for its role in the central executive of working memory [40] and top-down voluntary modulation of positive and negative emotions [41, 42]. Given that the decreased activation in the left DLPFC was consistent even during the neutral task in this study, abnormalities in this region is more likely to associate with difficulties in active cognitive control, i.e., cognitive manipulation in mental calculation. This inference was borne out of the behavioral results which showed significantly lower accuracy in patients relative to healthy subjects. The precuneus, serving as a component of the default mode network (DMN) which is always deactivated during goal-oriented activities, is particularly critical for the facilitation of self-referential cognitive activity and autobiographical memory [42, 43]. It has been evidenced that the self-projection related to personal past experience relies closely on the precuneus [43]. The relatively greater deactivation in the precuneus might imply the endeavor to suppress depressive rumination while MDD patients attempted to control their attention. The general pattern of the hypoactivity across task conditions indicated the poor executive control and maladaptive rumination, especially difficulties in shifting from DMN activity to task-positive network activity in MDD patients while participating in distraction tasks [44].

Additional regions with decreased activation in only the positive task were observed in the bilateral thalamus extending to the caudate, putamen, pallidum, and other subcortical areas. These regions are of importance to perception of pleasure and euphoria, owing to the close connection to the dopaminergic mesolimbic pathway [45]. Subcortical areas with reduced activation overlapped regions for reward anticipation and receipt, which replicated findings of previous studies [19, 46]. Recent studies indicate that striatal blunting also constitutes a risk factor for MDD, since reduced reward-related striatal activation was found in never-depressed youth who have a family history of MDD [47]. Hypoactivity in subcortical regions was found only for the positive task condition in the present study, indicating that abnormalities in reward processing are central to the pathophysiology of MDD.

### Impaired salience responses to positive stimuli in MDD patients

In accordance with prior studies, the co-occurrence of impaired reward processing and reduced activation in the salience network was identified in MDD patients [21, 48]. In and only in the positive condition, relatively decreased activation was found in the bilateral AI (or orbital insular cortices) and dorsal part of the ACC which constitute the salience network [25]. It has been built that the insula is situated at the interface of the cognitive, homeostatic, and affective systems of the human brain, providing a link between stimulus-driven processing and brain regions involved in monitoring the internal milieu [49]. The dACC underlies the interoceptive-autonomic processing and modules for sympathetic efference and interoceptive feedback processing [50, 51]. These regions also coactivate when perceiving varied forms of affective salience, such as enjoyable “chills” to music [52], faces of loved ones [53], and social rejection [54]. When allocating the attention from viewing pictures to arithmetic problems, multiple salient targets are supposed to elicit more increased activation in the salience-related regions. However, positive pictures failed to induce activation in the bilateral insulae of MDD patients, even when the corresponding activation could be elicited by neutral pictures (see S2 Fig). The bilateral insulae ought to be activated during tasks in response to the visually displayed novel stimuli, unless the significance of the stimuli is “insufficient” to be recognized by the salience system. These results replicate the findings in previous studies [14], and imply the impaired incentive salience processing in MDD patients.

To examine whether the alteration in the salience system of MDD patients is dependent on the specific task design or if it is more generalized for underlying the depressive pathology, we compared the standardized ALFF (zALFF) between groups based on their spontaneous neural fluctuations. Interestingly, increased zALFF values were observed in MDD patients, and mirrored the abnormalities revealed by the positive task to a large extent, situated at task-related, DMN, and salience-related areas, particularly at the bilateral AI. Aberrant increases in ALFF have been associated with depression in the prior studies [55]. This type of index was reported to correlate with baseline cerebral blood flow (CBF) that is typically coupled to brain metabolism [56]. Revealed by PET and fMRI, changes in regional CBF have been shown to be comparable to changes in regional glucose metabolism in response to task activation [57]. Mayberg discovered that CBF in healthy subjects increased in ventral paralimbic regions (e.g., AI and sgACC) and decreased in dorsal neocortical regions (e.g., prefrontal cortex, inferior parietal regions, dACC) when a sad mood state was induced and maintained. Furthermore, this triggered experience of sadness in healthy subjects matched the resting state of depressed patients [58], indicating that the overactive fluctuations of MDD patients during resting state might be associated with the immersion in negative emotions as well as the absence of pleasure. Therefore, it is likely that abnormalities in the bilateral AI observed in the present study are implicated in the increased vigilance towards negative valence and reduced sensitivity to positive valence [20, 21], due to the mood-congruent biases that are among the most robust research findings in neuropsychological studies of depression [59].

In other words, the depressive disorder exaggerated the amplitude of spontaneous oscillation in the bilateral AI of patients, and thus impacted on not only the intrinsic neural activity but also the way the brain responded to the tasks. The observation of the morbid intrinsic neural fluctuations in the bilateral AI rather than other reward-related areas suggests a predominate role of the impaired salience processing in the pathology of depression.

### Impaired dynamic switching between DMN and CEN in MDD patients

The salience network is conceived as a toggle system allowing mental switching between the DMN and central executive network (CEN) for processing self-referential and environmental information, respectively. The neurobiological basis is that, the AI and ACC which are rich in the von Economo neurons (VENs) are uniquely positioned to initiate control signals that activate the CEN and deactivate the DMN [24]. In healthy cohorts, the bilateral AI exhibit positive correlations with the CEN and reward circuit, and negative correlations with the DMN (see Fig 3). This fixed pattern of interaction between networks regulates the balance between the CEN and DMN, when individuals confront different situations. Nevertheless, MDD disintegrates the salience network, and alters the balance between CEN and DMN. Increased salience network dysfunction might lead to characteristic symptoms of depression, such as rumination (DMN), emotional over-reactivity (salience network), and emotional disinhibition (CEN) [55, 60]. In our study, reduced functional connectivity was found between the dACC and bilateral AI which probably induced difficulties during salience processing in monitoring error and conflict associated with the classical function of dACC [25, 61]. Furthermore, decreased connectivity between the left AI and its right counterpart was demonstrated, eliciting functional dissociation between the two critical nodes.

A tendency of functional reversion was observed in the left AI which showed relatively increased connectivity with the midline regions of the prefrontal and parietal cortices (e.g., mPFC, sgACC, PCC), while exhibiting decreased connectivity with regions anchored in the CEN and reward circuit (e.g., DLPFC, NAcc). Particularly, the morbid connectivity linking the left AI and sgACC presented significant positive correlation to the severity in loss of interests or pleasure; moreover, the decrease in the connectivity between the left AI and putamen positively correlated to the reduced BOLD responses in the left AI during the positive task (as shown in Fig 4). These results demonstrated the interaction of the impaired resting-state network and progression of depressive symptoms, as well as the synchronized alterations between spontaneous fluctuations and stimulus-driven responses in brains of MDD patients. On the other hand, reduced functional connectivity linking both the CEN (e.g., DLPFC, SMA) and the DMN (PCC, AG) was found in the right AI, demonstrating its weakened role for switching the two networks. However, connections between the right AI and bilateral lingual gyri (LG) were strikingly enhanced. The LG has been reported to be involved in the visual recognition network. The aberrant functioning in the LG is believed to be associated with the abnormal perception of emotions [62].

Our findings indicated chaotic spontaneous activity in the regions related to the left AI, and increased deactivation prone to excessive introspection in the regions connected to right AI in MDD patients. The aberrant intrinsic activity shows grounds for the malfunction of the salience system for processing task conditions, especially the positive condition, since coherent spontaneous fluctuations in human brain activity account for a significant fraction of the variability in measured task-evoked BOLD responses [63]. Consequently, patients with lower BOLD signal changes during the positive task in the right AI exhibited more severe MDD symptoms (as shown in Fig 4). We speculate that theses functional alterations gave rise to abnormalities in the salience detection of positive stimuli, and finally led to the diminished pleasure in MDD patients. Possible omission in shifting attention from viewing positive pictures to distractors, which arises from selective neglect of positive salience, might elicit less brain activation in the salience network and reward circuit. The absent perception of positive stimuli could also be likely to underlie the anhedonia, which is considered as a hallmark symptom of MDD. However, findings revealed by the current study are largely preliminary due to the small sample size. Further investigations with a large population are necessary to verify our speculation as well as whether the salience processing-related brain regions are possible markers for evaluating anti-depressive treatment in the future.

## Conclusions

In the present study, concurrent deficits in arousing activation were observed in both the reward circuit and salience network consisting of the dACC and bilateral AI during the positive task in only the MDD group. Subsequent ALFF analyses based on resting-state data showed abnormalities in the bilateral AI as well, revealing design-independent, generalized alterations in the salience network of MDD patients. Resting-state functional connectivity analyses were performed to explore neural mechanisms underpinning the aberrant salience processing, which showed a disrupted salience system in MDD patients and indicated patients’ difficulties in regulating the balance between CEN and DMN due to altered connectivity among the three networks. These results demonstrate impaired salience processing and resulting alterations in response to positive stimuli in MDD patients. Also, brain abnormalities synchronized across functional states in MDD patients can be evidenced by combination of task and resting-state fMRI analyses.

## Supporting Information

S1 Fig Results of group-level analysis based on factorial design. Significant main effects of group and condition, as well as significant interaction between group and condition were exhibited. Thresholds were set at a corrected p < 0.05 (voxel-level p < 0.005) as determined by AlphaSim correction.
(DOCX)

S2 Fig Results of contrasts between each task condition and baseline. Allocating attention from viewing pictures to arithmetic problems elicited more increased activation in the salience-related regions across the three conditions in healthy control (HC) subjects. While positive pictures failed to induce activation in the bilateral insulae of patients with major depressive disorder (MDD), even when the corresponding activation could be elicited by neutral pictures. The intervals between any two task blocks served as the baseline.
(DOCX)
