# Supplementary figures and images for: Task and Resting-State fMRI Reveal Altered Salience Responses to Positive Stimuli in Patients with Major Depressive Disorder
Source: PLoS One. 2016 May 18;11(5):e0155092. doi: 10.1371/journal.pone.0155092 (PMC4871416; doi:10.1371/journal.pone.0155092)

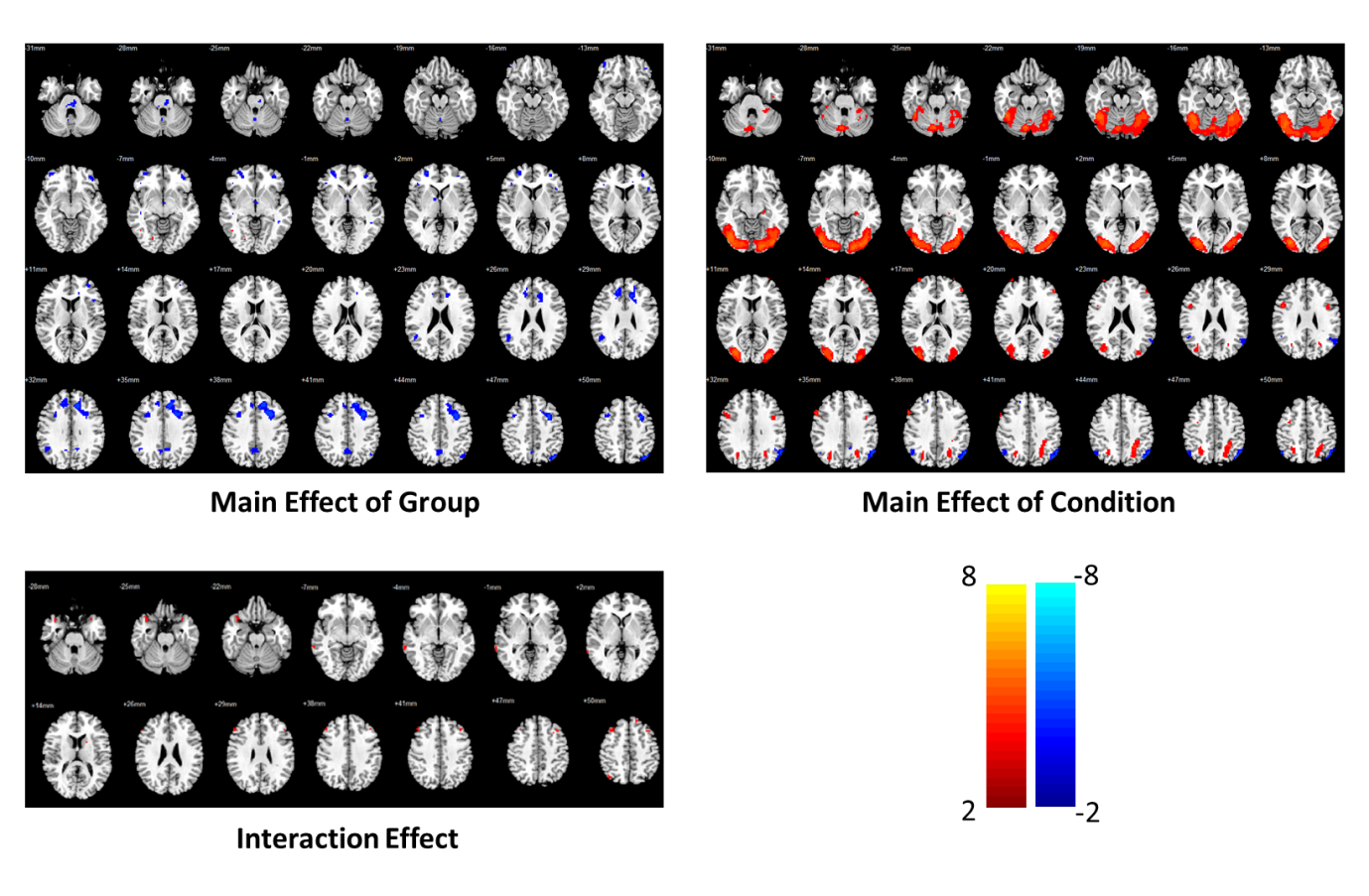


**S1 Fig. Results of group-level analysis based on factorial design**

Supplement: S1 Fig — Significant main effects of group and condition, as well as significant interaction between group and condition were exhibited. Thresholds were set at a corrected p < 0.05 (voxel-level p < 0.005) as determined by AlphaSim correction. (DOCX) [file pone.0155092.s001.docx]

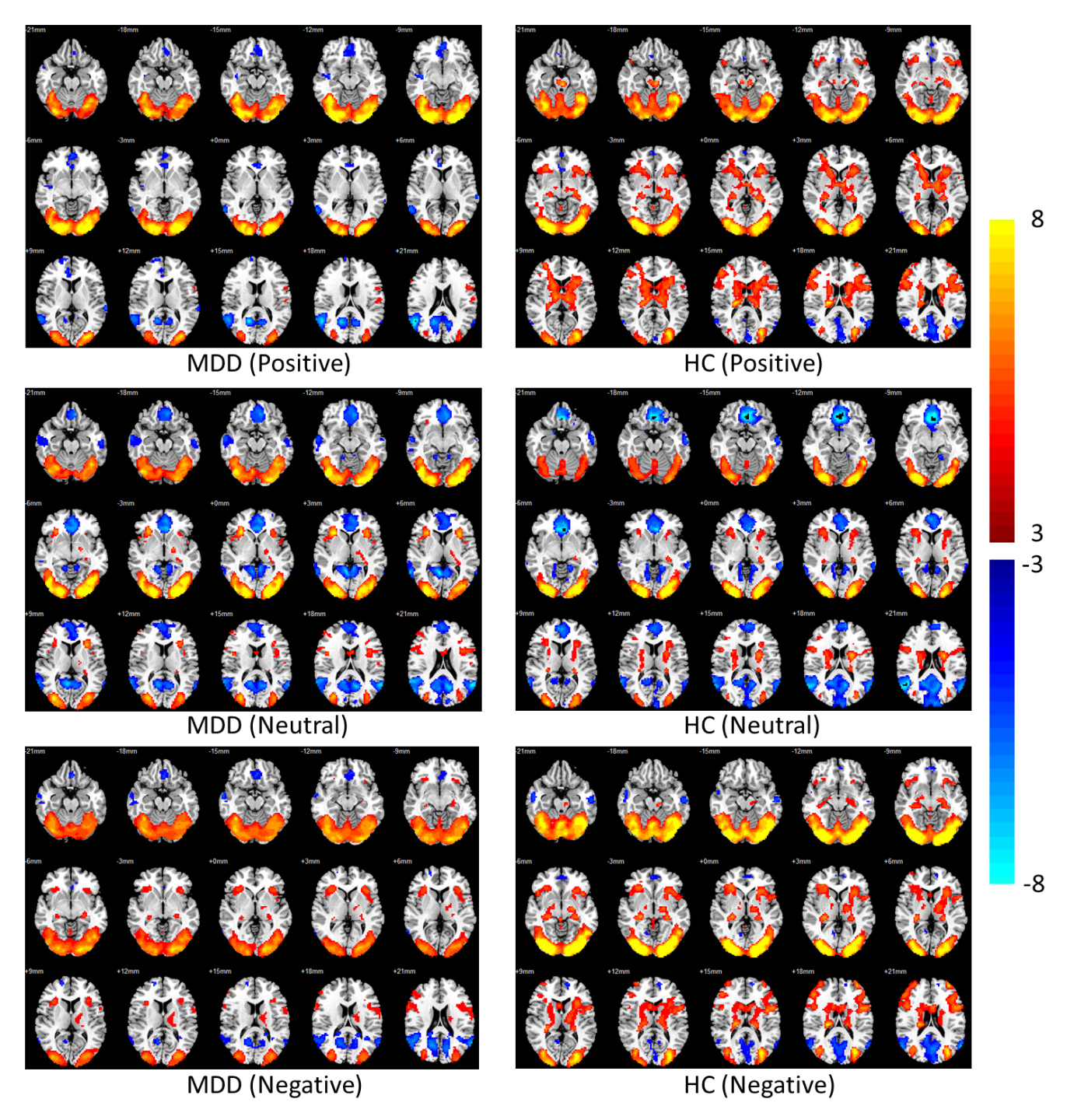


**S2 Fig. Results of contrasts between each task condition and baseline**

Supplement: S2 Fig — Allocating attention from viewing pictures to arithmetic problems elicited more increased activation in the salience-related regions across the three conditions in healthy control (HC) subjects. While positive pictures failed to induce activation in the bilateral insulae of patients with major depressive disorder (MDD), even when the corresponding activation could be elicited by neutral pictures. The intervals between any two task blocks served as the baseline. (DOCX) [file pone.0155092.s002.docx]
